# Chemical and Mechanical Evolution of a Volumetric Additive Manufacturing (VAM) Resin Under High-Temperature Storage

**DOI:** 10.3390/polym18101213

**Published:** 2026-05-16

**Authors:** Seyyed Kaveh Hedayati, Hossein Safari Mozajin, Kristoffer Almdal, Hans Nørgaard Hansen, Aminul Islam

**Affiliations:** 1 Department of Civil and Mechanical Engineering, Technical University of Denmark, 2800 Kgs. Lyngby, Denmark; skahe@dtu.dk (S.K.H.);; 2 Department of Chemistry, Technical University of Denmark, 2800 Kgs. Lyngby, Denmark; 3 Centre for Acoustic-Mechanical Microsystems (CAMM), Technical University of Denmark, 2800 Kgs. Lyngby, Denmark

**Keywords:** volumetric additive manufacturing, photopolymer resin, thermal aging, resin stability, oxygen inhibition, mechanical properties

## Abstract

Volumetric additive manufacturing (VAM) is a rapid, layerless photopolymerization process that fabricates three-dimensional (3D) objects by accumulating a projected light dose within a rotating resin vial. Since VAM relies on a polymerization threshold, small changes in resin chemistry, optical attenuation, or inhibition behavior may affect print fidelity and mechanical performance. However, the influence of resin storage history on VAM process stability remains insufficiently understood. This study investigates the chemical, optical, rheological, dimensional, and mechanical evolution of a representative acrylate-based VAM resin subjected to accelerated thermal aging. Resin samples were stored at 50 °C for 6, 12, and 48 days and compared with a non-aged resin. The aged resins were characterized, and the mechanical performance of printed and cast specimens was tested. The results indicate that storage did not cause any observable changes in molecular weight, and viscosity variations remained limited. However, aging produced measurable changes in optical, dimensional, and mechanical properties. The printed cylinder diameter increased from 12.9 mm for the non-aged resin to 14.4 mm after 48 days of aging. The tensile strength of printed samples peaked after 12 days of aging, and the compressive modulus increased with prolonged aging. Resin aging should be treated as an explicit manufacturing input, and routine resin monitoring and exposure recalibration are recommended to improve VAM reproducibility.

## 1. Introduction

Photopolymerization-based additive manufacturing (AM) methods, including stereolithography (SLA) and digital light processing (DLP), have emerged as versatile approaches for fabricating complex structures with high resolution [1]. Since the introduction of SLA, the paradigm of solidifying liquid photopolymer resins by localized light exposure has evolved from point-by-point laser scanning to the planar projection of entire two-dimensional (2D) layers using digital micromirror devices (DMDs) or liquid crystal displays (LCDs) [2]. Although these techniques reduce build time compared with point-wise scanning, they are still limited by sequential layer formation. As a result, parts are fabricated layer by layer, which can limit throughput, increase the need for support structures, and introduce anisotropic mechanical behavior [3].

Volumetric Additive Manufacturing (VAM) is a rapid, layerless photopolymerization process that addresses several limitations of conventional AM methods. By separating fabrication time from geometrical complexity, VAM solidifies three-dimensional (3D) objects within seconds to minutes by delivering a spatially controlled light dose into a photosensitive resin from multiple angles. The process is derived from computed tomography (CT) performed in reverse, in which a series of dynamically computed 2D projections is delivered through a rotating resin volume [4]. As the vial rotates, the accumulated dose exceeds the gelation threshold only within the target geometry. Since the object is generated volumetrically rather than by repeated layer-by-layer steps, VAM can reduce support requirements, avoid layer staircasing, and expand the range of materials and geometries that can be processed [2,5,6,7].

Recent studies have advanced VAM from an initial proof-of-concept process toward a more controlled manufacturing platform. Early demonstrations introduced tomographic VAM of acrylate-based resins and hydrogels to print complex centimeter-scale structures within short exposure times [6,7,8]. Later studies improved projection calculation, optical modeling, dual-color control, holographic tomographic printing, and process scalability [4,9,10,11,12]. These works show that VAM is not controlled only by the computed projection patterns, but also by the coupled interaction between optical attenuation, resin chemistry, photoinitiator efficiency, oxygen inhibition, heat generation, and dose accumulation.

Early demonstrations of VAM have successfully processed a range of photopolymers, although acrylate- and methacrylate-based formulations dominate many tomographic applications [2,5,9]. The successful execution of this process depends mainly on a nonlinear photopolymerization response [13]. Since in VAM the entire resin volume is exposed rather than only the voxels belonging to the part, selective solidification requires threshold conversion behavior. If polymerization occurs linearly with accumulated dose, off-target regions would also convert, eventually leading to loss of feature fidelity or even bulk polymerization [13,14]. Furthermore, in our previous study, we demonstrated that projection intensity and accumulated light dose directly control the extent of over-polymerization, and can significantly affect dimensional accuracy, edge sharpness, and surface roughness of fabricated parts [15].

To control over-polymerization in unwanted regions, radical propagation must be controlled and inhibited. Dissolved oxygen in acrylate and methacrylate resins provides this chemically thresholding response [14,16]. Oxygen reacts rapidly with carbon-centered radicals to form relatively low-reactivity peroxyl radicals, retarding propagation until the local oxygen concentration falls below a critical level [14,16]. This oxygen-inhibition period precedes the autoaccelerating gelation regime and is a key determinant of spatial dose contrast in VAM [13,14]. Recent work has further shown that oxygen inhibition in (meth)acrylate TVAM is not only a limitation, but also part of the thresholding mechanism required for selective solidification. However, the same mechanism makes print quality sensitive to photoinitiator concentration, light penetration, oxygen diffusion, and local dose distribution [14,17]. Therefore, oxygen must be considered both as a useful inhibitor and as a source of process sensitivity.

The role of oxygen becomes even more important when the resin is stored or thermally treated before printing. Recent studies on TVAM have shown that oxygen depletion and oxygen diffusion can limit resolution and print fidelity, especially in low-photoinitiator formulations where sufficient light penetration must be preserved [14,17]. Other studies have shown that thermal preparation and cooling can generate oxygen gradients inside the resin volume, since heating can reduce dissolved oxygen while cooling allows oxygen to diffuse back from the air–resin interface [18]. Mechanical handling strategies, such as minimizing headspace or eliminating the air–resin interface, can therefore affect the oxygen state before printing [18].

Despite its rapid fabrication capability, one of the major concerns in VAM is the storage stability of the highly reactive liquid resin. Recent studies emphasize that VAM performance is sensitive to formulation variables such as resin reactivity, inhibition, and viscosity, since these parameters together determine whether a sharp gelation threshold can be achieved during tomographic exposure [5,9]. In addition, process-control studies have shown that the correct exposure time in tomographic VAM can vary significantly, which means that a projection condition optimized for a fresh resin may not remain optimal after storage or reuse [11]. Accordingly, even small variations in inhibitor concentration or dissolved oxygen may perturb the polymerization threshold on which VAM depends [13,14,19].

To date, the mechanisms and extent of resin degradation in VAM remain insufficiently understood [5,9]. This gap is important since tomographic printing relies on accurate 3D dose accumulation within a nominally stationary resin. In this study, we experimentally investigate aging in a representative acrylate-based VAM resin using an accelerated aging protocol designed to mimic long-term storage and determine how storage-induced changes lead to geometric and performance variations. These experiments represent a practical scenario in which a resin bottle or printing vial is stored for months and then reused in laboratory or industry. The novelty of this work is that resin aging is treated not only as a shelf-life concern but also as a manufacturing input variable.

## 2. Materials and Methods

### 2.1. Materials and Resin Formulation

The VAM resin used in the experiment comprised poly(ethylene glycol) diacrylate (PEGDA, *M*_*n*_700 g mol^−1^; CAS: 26570-48-9) and bisphenol-A glycerolate diacrylate (BPAGDA; CAS: 4687-94-9) at a 1:1 (*w*/*w*) ratio. Camphorquinone (CQ)/ethyl 4-dimethylaminobenzoate (EDAB) (0.075/0.15 wt.%) were used as the photoinitiator (PI) and co-initiator. The commercial PEGDA/BPAGDA contained methoxy hydroquinone (MEHQ) and/or butylhydroxytoluene (BHT) as polymerization inhibitors. Although the experiments used PEGDA/BPAGDA, the results are relevant to any acrylate-based resin for VAM, highlighting general process-stability principles for volumetric photopolymerization. All chemicals were purchased from Sigma–Aldrich (Darmstadt, Germany) and used as received without further purification.

### 2.2. Accelerated Aging Protocol

To simulate long-term resin storage, an accelerated thermal-aging protocol was implemented. Four 250 mL portions of the resin were dispensed into amber DURAN borosilicate glass bottles, which were sealed to exclude ambient light and remained unopened for the entirety of their respective aging durations. The control sample (*Aged_0d*) was stored at room temperature (25 °C) without any thermal storage, whereas the remaining resin samples were stored under isothermal conditions at 50 ± 1 °C for 6, 12, and 48 days.

Equivalent storage time at room temperature was estimated using the *Q*_10_ model. In this model, the acceleration factor (*AF*) is defined as the ratio of the reaction rate at the elevated temperature, *k*(T) to that at the reference temperature, $k(T_{\mathrm{ref}})$ [20]:

$$\mathrm{AF}=\frac{k(T)}{k(T_{\mathrm{ref}})}=Q_{10}^{\left(\frac{T-T_{\mathrm{ref}}}{10}\right)}$$

The temperature coefficient (*Q*_10_) was assumed to be 2, as this approximation has been shown to agree closely with the Arrhenius model over the temperature range of 25 °C to 70 °C [20]. Accordingly, the *Q*_10_ method was used as a practical alternative to Arrhenius-based kinetic modeling, eliminating the need to determine the activation energy of the resin.

The acceleration factor was calculated as follows:

$$\mathrm{AF}=2^{\left(\frac{50-25}{10}\right)}=2^{2.5}\approx 5.66$$

The aged samples correspond to approximately 34, 68, and 272 days of equivalent storage at room temperature using the *Q*_10_ = 2 approximation. However, because the activation energy of the specific resin-aging process was not experimentally determined, these values should be interpreted as estimates of relative aging severity rather than exact kinetic equivalences. The *Q*_10_ approach is a phenomenological approximation and has been shown to agree best with Arrhenius-type behavior near a reference temperature of 25 °C, while deviations may occur when the apparent activation energy or reference temperature differs [20,21]. To evaluate the uncertainty associated with this approximation, an Arrhenius-based sensitivity analysis was performed [20]:

$${\mathrm{AF}}_{\mathrm{Arrhenius}}=\exp\left[\frac{E_{a}}{R}\left(\frac{1}{T_{\mathrm{ref}}}-\frac{1}{T}\right)\right]$$

where $E_{a}$ is the apparent activation energy, *R* is the gas constant, $T_{\mathrm{ref}}=298.15\;K$, and T=323.15K. The $Q_{10}=2$ acceleration factor of 5.66 corresponds to an apparent activation energy of approximately 55.5 kJ mol^−1^, or 0.575 eV. Using apparent activation energies of 0.59–0.67 eV discussed for polymer thermal-aging estimates [20], the Arrhenius base equivalent storage times for 6, 12, and 48 days at 50 °C are approximately 35.5–45.1, 70.9–90.2, and 283.7–361.0 days at 25 °C, respectively. This corresponds to an uncertainty of approximately +4.5% to +32.9% relative to the *Q*_10_ estimate. Therefore, the accelerated-aging protocol is used here as a comparative framework for evaluating storage-induced trends, not for an absolute shelf-life prediction.

Throughout the thermal storage, the bottles remained sealed, unopened, and protected from ambient light. After the predefined periods, the bottle was removed from the oven and handled according to a strict protocol. Immediately after removal from the oven, the bottle was opened, and the hot resin was transferred directly into VAM printing vials. Each test tube was filled to the top to minimize air headspace, then sealed. The filled and sealed test tubes were allowed to cool to room temperature, then printed immediately afterward.

### 2.3. VAM Setup and Software

**Figure 1:**
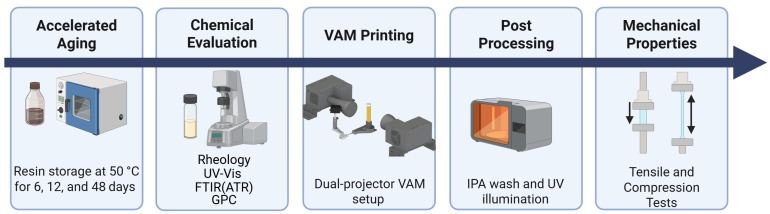
Experimental concept and workflow for evaluating thermal aging effects.

Figure 1 shows the experimental concept and workflow for evaluating thermal aging effects. Two custom-built 1080P DLP projectors (TVP07, Xiamen Zhisen, Xiamen, Fujian, China), each providing a projection area of 32.4 × 57.6 mm^2^ and facing each other, were used as light sources to illuminate upper and lower sections of the resin container. An in-house developed software, TomoPrint, generated the 2D light patterns required for Virtual Stitching of Coordinated Projections as described in our previous work [22]. In summary, the software uses OpenGL compute shaders to perform parallel ray generation and traversal to accurately simulate physical optical interactions, such as refraction at container interfaces and volumetric beam attenuation within the resin. To achieve a uniform 3D energy distribution and resolve undesired dose accumulation from overlapping projections, the pixel intensities of the projections are iteratively adjusted using an object-space model optimization (OSMO) algorithm [10]. A summary of the VAM process parameters and the intensity calibration procedure for the dual-projector is provided in Appendix A.1. The upscaled VAM printing process is shown in Video S1.

### 2.4. Post-Processing Procedure

For post-processing, uncured resin was drained from the test tube. The printed part was immersed in isopropyl alcohol (IPA) for 6 min to remove residual resin, followed by multiple rinses with IPA, and subjected to a brief UV exposure (“pre-hardening”) for 10 s. The sample was removed and dried under ambient conditions for one hour. Final polymerization was achieved by 33 min of light projection using an Otoflash G171 light-curing unit (Dentona GmbH, Dortmund, Germany), followed by exposure to a 470 nm light source (SOLIS-470C, Thorlabs, Mölndal, Sweden) for 20 min, both under a nitrogen environment.

### 2.5. Chemical and Rheological Characterization

UV–Visible spectra were collected in the interval of 400 to 700 nm (Agilent G1103A spectrophotometer, Santa Clara, CA, USA; 10 mm path length PMMA cuvette) to evaluate the absorption properties of the photoresin and to monitor PI evolution during aging. Real-time spectra were acquired in attenuated total reflectance mode (diamond ATR). For each run, an unilluminated resin droplet served as *t* = 0. In situ polymerization was driven by a blue LED (465 nm) operated at 10 mW cm^−2^ at the sample plane using an intermittent dosing protocol: 4 s illumination, immediate spectrum acquisition, and repetition for 30 cycles. Double-bond conversion was determined by monitoring the acrylate C=C stretching peak at 1638 cm^−1^, normalized to the aromatic ring vibration at 1610 cm^−1^, which served as an internal non-reactive reference band. At each point *t*, the corresponding peak heights were used to calculate the ratio: $R\left(t\right)=A_{1638}\left(t\right)/A_{1610}\left(t\right)$, with R(0) obtained from the unilluminated resin. The double-bond conversion (DBC) was then obtained as follows [23]:

$$\mathrm{DBC}\left(t\right)=\left[1-\frac{R(t)}{R(0)}\right]\times 100\%$$

Molecular weight ($M_{n}$, $M_{w}$, and dispersity) of fresh and aged resins was determined by gel permeation chromatography (GPC) using an Agilent 1260 Infinity II GPC/SEC system (Agilent Technologies, Santa Clara, CA, USA). Separations were performed at 30 °C with chloroform flowing at 1.0 mL min^−1^. Column calibration employed narrow-dispersity polystyrene standards. The resin viscosity was measured using a Discovery HR-2 Hybrid rheometer (TA Instruments, New Castle, DE, USA) using 40 mm diameter parallel plates at a gap of 500 μm and shear rate ($\dot{\gamma }$) of 1 to 100 s^−1^ at 25 °C.

### 2.6. Mechanical Testing

Mechanical tests were performed using an Instron 3400 Series universal testing machine (Instron, Norwood, MA, USA). Tensile specimens were prepared using the upscaled VAM setup and tested according to ASTM D638 Type V specimens [24]. At least five specimens were tested for each aging condition. The tests were conducted at a crosshead speed of 2 mm/min using a 20 kN load cell. The tensile modulus was calculated from the linear elastic region of the stress–strain curve, and the ultimate tensile strength (UTS) was determined as the maximum stress recorded before specimen failure.

Compression testing was performed according to ASTM D695 [25]. Cylindrical specimens with a nominal diameter of 12.7 mm and a height of 25.4 mm were printed for each aging condition. Before testing, the diameter and height of each specimen were measured using a digital caliper and used to calculate the stress. At least five specimens were tested for each group. Compression testing was conducted at 1 mm/min using a 50 kN load cell. Since no fracture occurred during compression testing, the compressive stress at 15% strain was used for comparison. The compressive modulus was calculated from the linear elastic region of the stress–strain curve.

In parallel, cast specimens were prepared as non-printed controls to evaluate the resin response under simplified curing conditions. Tensile specimens were prepared by casting the resin in silicone rubber molds and curing with a 470 nm LED source at irradiances of 10, 70, and 110 mW/cm^2^. The corresponding illumination times are provided in Table A4. Cylindrical compression specimens were cast and cured at an irradiance of 28 mW/cm^2^ for 40 s. All cast specimens were subjected to the same post-processing procedure used for the VAM-printed specimens. Data are reported as mean ± standard deviation.

## 3. Results

### 3.1. Chemistry and Optics of the Resin

**Figure 2:**
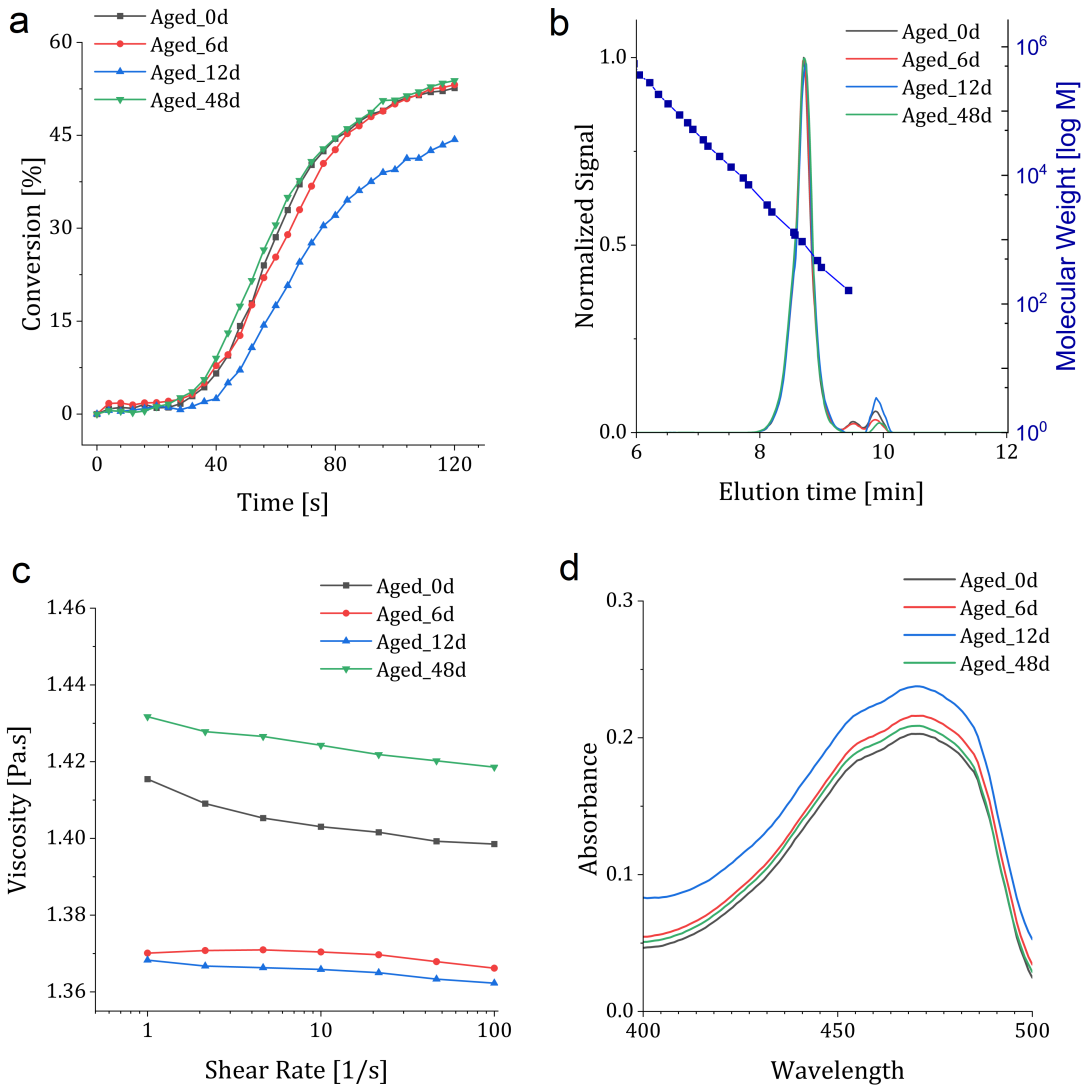
Optical and physicochemical stability of the resin under high-temperature storage: (**a**) Real-time conversion vs. time, (**b**) GPC chromatograms, (**c**) steady-shear viscosity (1–100 s^−1^), (**d**) UV–vis spectra.

Real-time FTIR can be used to track any kinetic variation within aged resin. According to the obtained results, all tested resins reached a high final DBC by 120 s, clustering between 52% and 54%. On the other hand, *Aged_12d* reached a lower value of 44% (Figure 2a). Aging mainly affected the rate and initiation time of the conversion. The fastest sample (*Aged_48d*) reached 50% conversion after 96 s and attained a conversion of 54% by 120 s, while the slowest sample (*Aged_12d*) did not reach 50% conversion within the experiment timeframe. *Aged_0d* and *Aged_6d* showed intermediate behavior. Also, conversion rates were calculated from linear fits to the mid-range slope between 44 and 80 s of the graph. Conversion rates of 1.01, 1.02, 0.94, and 0.90% s^−1^ were measured for *Aged_0d*, *Aged_6d*, *Aged_12d*, and *Aged_48d*, respectively. The DBC results showed efficient photopolymerization and high conversion rates among all storage conditions, although aging had a modest effect on the polymerization kinetics. FTIR spectra of aged, non-illuminated resins were compared with those of the *Aged_0d* resin in order to evaluate any possible dark polymerization during storage. The measured dark-polymerization values remained within ±1.5% across all aging conditions, indicating that no detectable dark polymerization occurred during storage, and if there was any change, it remained below the sensitivity of the measurement. It should also be considered that the ATR-FTIR experiment was performed on a thin layer of resin exposed to air. In this condition, atmospheric oxygen can diffuse back into the resin more rapidly than in the larger sealed volume used for printing. As a result, this may partially mask the extent of oxygen depletion that occurred during storage. Therefore, the FTIR results may show smaller changes in the induction period than the printed parts, where the resin volume was larger, and oxygen replenishment before exposure was more limited.

Figure 2b presents the GPC results of the resins. To minimize variations related to concentration effects, each chromatogram was normalized prior to analysis. The normalized results showed a single dominant distribution across all aged resins, centered at approximately 8.7–8.8 min. Only minor changes in peak position and in the late-eluting tail were observed during storage. To further evaluate these changes in profile shape, the normalized signal was integrated over four contiguous time windows spanning 8–9 min. Aging caused a gradual redistribution of the elution profile, characterized by a slight shift in the peak maximum and an increase in the late-eluting fraction, 8.75–9.00 min, of 16.2% for *Aged_48d* and 15.1% for *Aged_12d* relative to the non-aged resin. No significant change was observed for *Aged_6d*. Detailed values across all storage conditions are provided in Appendix A.2 Table A2.

The limited variation indicates that high-temperature storage did not cause any major changes in molecular weight detectable by GPC. Therefore, the mechanical and dimensional variations observed after printing cannot be attributed to dark polymerization, degradation, or oligomerization of the resin. However, GPC does not describe the final crosslinked network formed during photopolymerization. Even similar resins before polymerization can still yield different mechanical properties.

Extending the comparison of resin characteristics, steady-shear rheometry over 1–100 s^−1^ was performed on the resins. According to the result, a uniform Newtonian response across all formulations (≤2%) was observed. At 100 s^−1^ shear rate, viscosities were measured to be 1.398, 1.366, 1.362, and 1.419 Pa·s for *Aged_0d*, *Aged_6d*, *Aged_12d*, and *Aged_48d*, respectively. Short-term aging resulted in a 2–3% decrease in viscosity compared to the *Aged_0d* sample. On the other hand, extended aging resulted in a nearly 2% increase with respect to *Aged_0d* (Figure 2c). Therefore, the rheology results indicate that no major viscosity-driven change in printability occurred during storage.

UV–Vis analysis was performed to evaluate optical attenuation variations in the aged resins (Figure 2d). At the printing wavelength of 460 nm, the *Aged_0d* sample showed an absorbance of 0.190. For the aged resins, absorbance increased to 0.203 for *Aged_6d* and 0.225 for *Aged_12d*, then decreased to 0.196 for *Aged_48d*. It is worth mentioning that no shift in the absorption band position was observed. The penetration depth at 460 nm was estimated from the Beer–Lambert relationship [6]:

$$D_{p}=\frac{1}{\alpha }=\frac{l}{\ln\left(10\right)A_{460}}$$

where $D_{p}$ is the penetration depth, α is the absorption coefficient, l=10mm is the cuvette path length, and $A_{460}$ is the absorbance at 460 nm. The calculated penetration depths were 22.9, 21.4, 19.3, and 22.2 mm for *Aged_0d*, *Aged_6d*, *Aged_12d*, and *Aged_48d*, respectively. Compared to the non-aged resin, the maximum reduction in penetration depth was approximately 15.5% for *Aged_12d*. The UV–Vis results show that aging can lead to measurable changes in optical attenuation, thereby affecting the spatial dose distribution during VAM.

Overall, the trends shown in Figure 2 indicate that thermal aging did not produce a single degradation pathway across all measured resin properties. Instead, the resin-state changes were non-uniform and depended on the characterization method. In this study, the chemical and physicochemical measurements serve as a resin-state screening framework for evaluating resin quality before printing. Each method targets a different possible source of resin instability. UV–Vis spectroscopy evaluates changes in optical attenuation at the printing wavelength and possible changes associated with photoinitiator depletion. Dark FTIR evaluates whether unwanted dark polymerization occurred during storage, while real-time FTIR tracks changes in induction behavior and polymerization kinetics under illumination. GPC monitors the molecular-weight distribution of the resin and possible oligomerization or degradation. Rheology provides a quality metric to determine whether storage caused major changes that could affect handling or printability.

### 3.2. Mechanical Evolution: Printed vs. Cast

**Figure 3:**
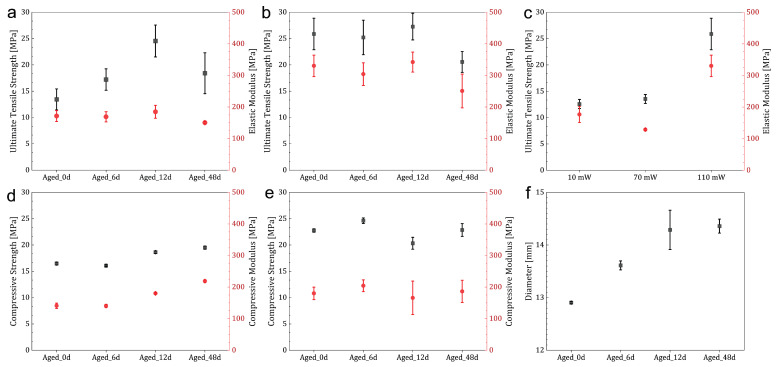
Mechanical performance of (**a**) Printed tensile, (**b**) Cast tensile, (**c**) Cast tensile with different light intensities, (**d**) Printed compressive, (**e**) Cast compressive, and (**f**) Diameter of the compression printed parts. Points/bars: mean ± Standard Deviation.

Figure 3a illustrates the tensile properties of the printed parts. Aging caused an approximately twofold increase in ultimate tensile strength (UTS) from *Aged_0d* to *Aged_12d*, followed by a partial decrease at *Aged_48d*. While the tensile modulus (*E*) remained within a relatively narrow range of 150–185 MPa. UTS increased from 13.4 MPa for *Aged_0d* to 24.5 MPa for *Aged_12d*, then decreased to 18.4 MPa for *Aged_48d*. Over the same aging interval, *E* variation was minimal and remained within the range of 170–185 MPa before decreasing to 150.5 MPa at *Aged_48d*. Although the upscaled dual-projector setup enabled fabrication of long tensile specimens, a visible stitching line remained detectable at the boundary between the projected fields. This line is associated with small local differences in dose accumulation, projector alignment, optical overlap, or pixel-intensity matching during the virtual stitching process [22]. However, local mechanical properties in the stitching region were not directly measured in the present study. No tensile fracture was observed along the stitching line, indicating that, under the tested conditions, this region was not the dominant failure site.

As shown in Figure 3b, the cast tensile specimens exhibited approximately twice the stiffness and strength of the printed specimens at *Aged_0d*, and this relationship was maintained at *Aged_6d* and *Aged_12d*. However, at *Aged_48d*, UTS and *E* decreased by 20% and 25%, respectively. Notably, the UTS of the *Aged_12d* printed sample approached that of the cast *Aged_0d* specimen, whereas *E* remained approximately 45% lower. In the cast tensile specimens, light intensity was a major factor affecting mechanical properties. According to the results, reducing the illumination intensity from 110 mW cm^−2^ to 70 mW cm^−2^ or 10 mW cm^−2^ resulted in an approximately 50% decrease in both UTS and *E*, as shown in Figure 3c. These results show the resin’s sensitivity to processing conditions, even in the absence of the geometric and stitching effects found in printed parts. To evaluate whether the mechanical variations could be explained by incomplete curing, the final DBC of the printed and cast parts was measured and compared. As detailed in the Appendix A (Table A3 and Table A4), ANOVA revealed no statistically significant differences in the final degree of conversion among groups (*p* > 0.05). Therefore, the observed mechanical property variations cannot be assigned only to changes in the average final DBC. However, DBC measured at selected locations represents an average chemical conversion and does not resolve spatial variations in network formation, local crosslink-density distribution, residual stress, or voxel-to-voxel connectivity. These factors may differ as a result of aging-induced changes in cure kinetics and light-dose threshold behavior, even when the final average DBC values are similar.

The compressive properties of the printed parts, shown in Figure 3d, also exhibited a clear aging-related improvement. The compressive modulus increased by 55% from *Aged_0d* to *Aged_48d*, with 28% of this increase already occurring at *Aged_12d*. The compressive modulus increased from 140.8 MPa for *Aged_0d* to 180.2 MPa for *Aged_12d* and 218.9 MPa for *Aged_48d*. Since no fracture occurred during compression testing, the stress at 15% strain ($\sigma_{15}$) was extracted and used for analysis and comparison. Compressive strength followed a similar trend, increasing by 18% at *Aged_48d*, indicating improvement in stiffness and load-bearing capacity at a fixed strain.

Similar to tensile parts, compressive cast parts showed a moderate response to aging. The compressive strength at 15% strain ($\sigma_{15}$) showed values of 22.76, 24.62, 20.33, and 22.85 MPa for *Aged_0d*, *Aged_6d*, *Aged_12d*, and *Aged_48d*, respectively. Similarly, the compressive modulus showed only minor variation, with values of 180.1, 204.3, 165.9, and 186.4 MPa for the same resins, respectively. No statistically significant differences were observed among the cast samples (one-way ANOVA, *p* > 0.05; Figure 3e). Compared with the printed specimens, only *Aged_12d* and *Aged_48d* reached strength values comparable to those of the cast parts.

**Figure A2:**
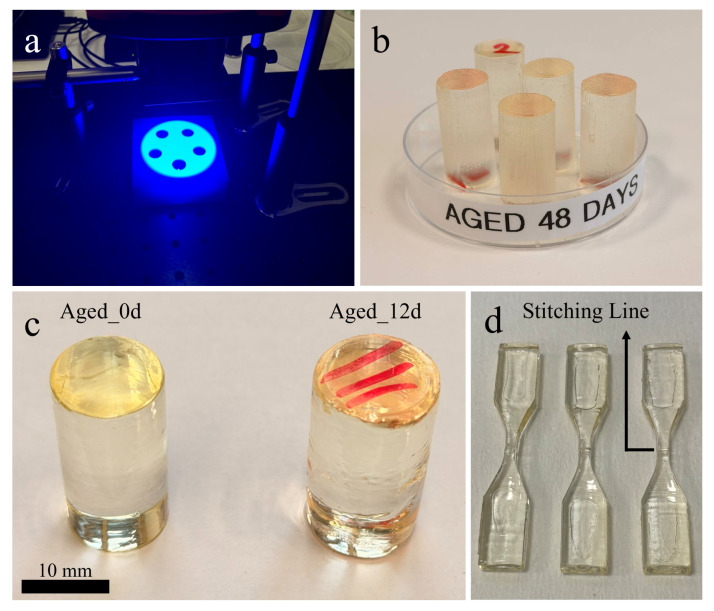
(**a**) Casting of the compression specimens. (**b**) Cast parts after post-processing. (**c**) Printed compression part with *Aged_0d* and *Aged_12d* resins. (**d**) Tensile specimens printed using the upscaled dual-projector setup.

Figure 3f shows the diameter of the compression printed parts. The printed parts’ diameters increased from 12.9 mm for *Aged_0d* to 14.4 mm for *Aged_48d* (Figure 3f). This 13% increase compared to the nominal targeted diameter indicates that aging altered the VAM printing process behavior and compromised dimensional fidelity (Figure A2).

## 4. Discussion

Accelerated thermal aging of the PEGDA/BPAGDA resin provides a workflow to separate the roles of chemistry, optics, and the inhibitor threshold in the printing process. In VAM, polymerization occurs when the accumulated dose exceeds an effective threshold governed by photoinitiation, oxygen inhibition, and optical properties. Storage may shift this threshold, which affects both feature dimensions and mechanical properties under fixed printing conditions. Several mechanisms may contribute to these kinetic changes. First, changes in dissolved oxygen availability could modify the oxygen-inhibition period and alter the effective polymerization threshold, since oxygen inhibition is known to influence acrylate and methacrylate photopolymerization and has been identified as an important thresholding mechanism in tomographic VAM [14,16,26]. Second, phenolic stabilizers such as MEHQ, which act synergistically with dissolved O_2_, may be partially consumed or redistributed during elevated-temperature storage [27,28]. However, dissolved O_2_ concentration and inhibitor concentration were not directly measured in the present study. These possible effects are consistent with the observed changes in cure kinetics, optical response, dimensional fidelity, and mechanical properties, but their individual contributions require direct quantification.

In VAM, even small changes in initiator–inhibitor balance or optical properties can lead to substantial differences in the spatial and temporal onset of polymerization. Previous work has shown that oxygen inhibition does not simply inhibit polymerization but instead helps define a polymerization threshold [14,16]. A reduction in available *O*_2_ can shift this threshold and expand the polymerized regions under the same projected dose. Aging also induced measurable UV–Vis absorbance changes, altering the optical penetration depth at the printing wavelength. The *Aged_12d* resin showed the highest absorbance at 460 nm and the lowest calculated penetration depth, indicating that the depth-dependent dose distribution was modified. However, the critical gelation dose was not directly measured in this study. Therefore, the optical changes should be considered alongside possible changes in oxygen inhibition, inhibitor activity, and polymerization kinetics when interpreting the observed increase in the diameter of the printed cylinders.

The mechanical property results are consistent with a non-monotonic change in cure kinetics and network formation during aging. The tensile strength of printed parts nearly doubled from *Aged_0d* to *Aged_12d*, whereas the tensile modulus remained within a narrower range. In contrast, the compressive modulus showed a greater increase with aging across all groups. Because all resins maintained Newtonian behavior and only small viscosity changes were observed, bulk viscosity is not considered the primary source of the observed printability and property variations. The higher tensile properties of *Aged_12d* are more likely related to a beneficial shift in the polymerization pathway under the fixed VAM printing parameters. Variations in inhibition, optical attenuation, and radical kinetics may improve voxel-to-voxel connectivity and tensile load transfer without significantly increasing stiffness. However, the decrease in tensile strength observed for *Aged_48d* cannot be explained by inhibitor depletion alone. Prolonged aging may alter the radical balance and enable earlier gelation, increasing local crosslink density and compressive stiffness while reducing tensile properties. These mechanisms could reduce tensile strength even when the final average DBC remains similar. The transfer protocol was designed to minimize oxygen re-equilibration before printing by transferring the hot resin directly into test tubes, followed by sealed cooling and immediate printing [18].

The comparison between printed and cast specimens should be interpreted cautiously. The cast samples isolate the resin response under a simpler polymerization condition, whereas the VAM-printed samples include additional process effects. Therefore, the printed and cast difference cannot be quantitatively separated into intrinsic material behavior and process-induced defects using the present dataset. Light intensity and generated heat during the casting process further modulate these effects. Higher intensity increases local dose and self-heating, lowers activation energy, enhances molecular mobility, and allows higher conversion before vitrification. On the other hand, lower intensity or stronger attenuation prolongs induction and limits final conversion [29,30]. The cast samples help distinguish intensity effects from high-temperature storage. In cast tensile parts, reducing the light intensity reduced both UTS and *E* by half. Faster initiation and higher reaction rates generally correspond to higher crosslink density and stiffer networks, up to the point at which heterogeneity begins to compromise toughness [29]. However, the cast *Aged_48d* specimens showed a 20% reduction in UTS and a 25% reduction in *E* relative to *Aged_0d* under similar printing process conditions. This behavior is consistent with a storage-induced kinetic shift that impairs tensile performance without substantially affecting compressive response. Bulk characterization shows limited chemical drift. In summary, GPC showed a dominant peak, rheology remained Newtonian, and UV–Vis revealed a minor modulation of blue-light absorbance. Even such small optical changes can disrupt feature formation in VAM, where printed parts’ fidelity depends on absorption and scattering of the resin.

Finally, the mechanical behavior can be linked to polymerization physics through oxygen quenching, induction behavior, and gelation history. Elevated-temperature aging may reduce dissolved *O*_2_ availability or alter inhibitor effectiveness, which could shorten the induction time and lower the effective energy required to initiate polymerization. Under the same projected light dose, such a shift can change the local gelation history and voxel connectivity, which is consistent with the higher tensile strength of printed *Aged_12d* samples and the increase in printed compressive modulus. In the *Aged_48d* condition, however, a faster approach to gelation may promote a less homogeneous network and reduce tensile robustness, even while compressive stiffness increases.

The presented results showed that fixed projection parameters can lead to substantial variations in both dimensions and mechanical properties. To overcome this, exposure conditions should be recalibrated after storage by monitoring absorbance at the projection wavelength and performing a calibration test. For instance, it can be implemented as a rapid dose sweep using a simple calibration geometry printed in the same vial to identify the required dose to recover the targeted dimension. Aging can be treated as a resin variable that needs to be tracked and recalibrated. In controlled cases, it may be used to tune mechanical response. In the present study, intermediate aging increased tensile strength, while extended aging increased compressive modulus but also increased feature size. Storage temperature and duration should also be recorded to ensure reproducibility, even when bulk chemistry appears stable. Resin aging can be treated not only as a shelf-life concern, but also as a repeatable process-calibration variable in VAM.

## 5. Conclusions

The successful execution of VAM relies on the seamless integration of optical, mechanical, computational, and chemical systems. Even a small deviation in any single domain can severely compromise print viability. Although hardware and algorithms are frequently optimized, the photopolymer resin itself remains a highly dynamic variable. Even for an optimally formulated resin, long-term storage may introduce kinetic instabilities that affect the final fabricated part in VAM. To address this issue, we evaluated how thermal storage history affects the process window, dimensional fidelity, and mechanical properties of resins used in VAM. An accelerated thermal storage protocol was applied, and resin evolution was assessed through chemical characterization alongside mechanical performance evaluations. Under fixed printing and post-processing conditions, substantial mechanical and dimensional changes were observed despite overlapping final DBC. Specifically, aging led to a 13% dimensional expansion in printed parts, a continuous increase in compressive modulus, and variations in ultimate tensile strength. These substantial mechanical and dimensional changes occurred even though the resin’s chemical properties did not vary significantly. These results indicate that final part properties depend not only on total conversion, but also on the network architecture and heterogeneity established by cure kinetics. Aging directly affects these polymerization kinetics and the oxygen-induced dose threshold, which ultimately control voxel connectivity and spatial dose distribution. Therefore, resin storage history must be treated as an explicit variable within the process-control methodology. The findings suggest the need for a simple control strategy for VAM, such as pre-print verification and exposure recalibration, to compensate for potential changes, maintain the process within the intended window, and stabilize part properties over the resin’s storage life. A limitation of this study is that dissolved O_2_ concentration and inhibitor depletion were inferred mechanistically rather than directly quantified. Therefore, future work should directly measure the dissolved O_2_ concentration and inhibitor depletion during storage and link these metrics to induction time and dose-threshold drift. Furthermore, a transferable calibration framework should be established to translate simple resin-state indicators into dose updates using a standard calibration geometry. This approach should be validated across various resin chemistries and photoinitiator systems to define general boundary conditions for VAM resin shelf life and process robustness.

## Figures and Tables

**Figure A1:**
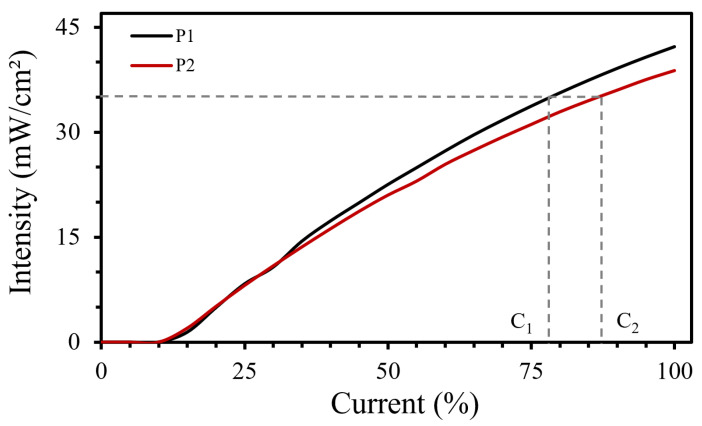
Measured optical power versus input current for both projectors.

**Figure A3:**
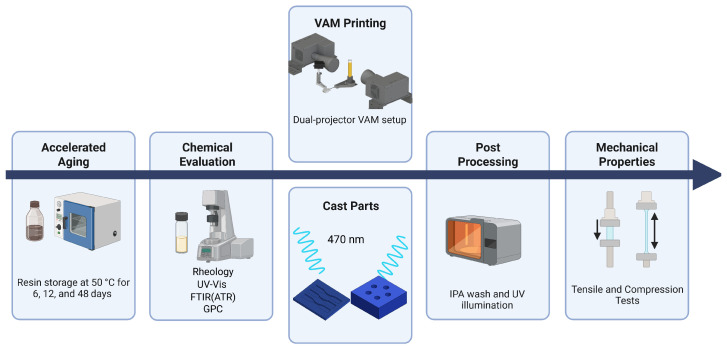
Experimental concept and workflow for evaluating thermal aging effects in VAM resins, including both printed and cast parts.

## Data Availability

The original contributions presented in this study are included in the article/Supplementary Material. Further inquiries can be directed to the corresponding author.

## Supplementary Materials

The following supporting information can be downloaded at: https://www.mdpi.com/article/10.3390/polym18101213/s1, Video S1: Upscaling Volumetric Additive Manufacturing (VAM) printing process.

## Abbreviations

The following abbreviations are used in this manuscript:

AM	Additive Manufacturing
VAM	Volumetric Additive Manufacturing
CAD	Computer-Aided Design
fps	Frames per second
CQ	Camphorquinone
EDAB	Ethyl 4-dimethylaminobenzoate
PEGDA	Poly(ethylene glycol) diacrylate
BPAGDA	Bisphenol-A glycerolate diacrylate
PI	Photoinitiator
DBC	Double-Bond Conversion

## Appendix A

### Appendix A.1. Volumetric Printing Parameters

Although both projectors look similar, there might be output power mismatches even with identical input current settings. To account for any potential variations, the optical power of each projector should be measured. Then, a calibration function in the code can be used to scale the output power and ensure that both projections have the same output intensity. Figure A1 shows the measured output power of the projectors with different input currents and how they were matched by modifying the input current. This promotes uniform part formation. Even small mismatches in light intensity can disrupt the dimensional fidelity and mechanical performance of printed parts.

Table A1 summarizes the VAM printing parameters used for fabrication of the compression and tensile specimens. These parameters include illumination intensities, frame rates, the number of rotations, and the total projection time required to reach the necessary polymerization threshold.

**Table A1:** Summary of the VAM parameters used for fabrication of compression and tensile specimens.

Parameter	Compression (Single Projector)	Tensile Specimens (Dual-Projector)
Illumination intensity	28 mW cm^−2^	35 mW cm^−2^
Number of projections	1200	720
Frame rate	60 fps	30 fps
Number of rotations (360°)	1	2
Total projection time	20 s	48 s

### Appendix A.2. GPC Analysis

GPC analysis was performed to assess molar-mass evolution in the resin formulations before and after thermal aging. The measurements were used to compare the number-average ($M_{n}$), weight-average ($M_{w}$), and z-average ($M_{z}$) molar masses, together with the polydispersity index (PDI), for the Aged_0d resin and the thermally aged resins after 6, 12, and 48 days of storage at 50 °C.

Table A2 presents the results of the GPC. High-temperature storage did not result in any measurable change in the overall molecular weight of the resins, indicating that no dark polymerization or oligomerization occurred. The only detectable effect of aging was a slight increase in PDI after 48 days, reaching approximately 1.23. These findings suggest that prolonged thermal exposure caused only limited molecular-weight broadening in the uncured resin. However, GPC does not characterize the insoluble crosslinked network formed after photopolymerization; therefore, the GPC results alone cannot explain or exclude changes in mechanical properties after curing.

**Table A2:** GPC results for the *Aged_0d* resin and thermally aged resins after 6, 12, and 48 days of storage at 50 °C.

Sample	$M_{n}$	$M_{w}$	$M_{z}$	PDI
*Aged_0d*	806	948	1140	∼1.18
*Aged_6d*	801	948	1149	∼1.18
*Aged_12d*	766	903	1130	∼1.18
*Aged_48d*	774	949	1198	∼1.23

### Appendix A.3. Real–Time FTIR Analysis

FTIR-ATR analysis was performed on cross-sections of printed and cast specimens after tensile testing. The center point of each specimen was used to determine the final double-bond conversion (DBC).

Table A3 and Table A4 summarize the results of the final DBC for both printed specimens and the cast controls. As shown, the printed specimens, regardless of storage conditions, achieved conversion levels comparable to those of the cast parts. One-way ANOVA was performed across the groups. The analysis confirmed that neither prolonged thermal aging of the printed parts nor the varying light intensities applied to the cast samples resulted in a statistically significant difference in the final degree of conversion (*p* > 0.05). It can be inferred that the dimensional and mechanical shifts observed in aged parts are not solely due to higher conversion rates.

**Table A3:** DBC of volumetrically printed tensile specimens after post-processing, reported as mean values and standard deviations for *Aged_0d*, *Aged_6d*, *Aged_12d*, and *Aged_48d*.

Resin	DBC [%]	SD [%]
*Aged_0d*	44.15	5.78
*Aged_6d*	46.28	5.79
*Aged_12d*	39.10	7.73
*Aged_48d*	38.00	8.90

**Table A4:** DBC of tensile cast specimens prepared from *Aged_0d* resin under different exposure intensities and projection times, reported as mean values ± standard deviation.

Exposure Condition	DBC ± SD [%]
10 mW, 20 s	45.88 ± 5.84
70 mW, 3 s	55.09 ± 1.99
70 mW, 20 s	53.34 ± 2.36
110 mW, 48 s	48.75 ± 9.63

### Appendix A.4. Casting of Tensile and Compression Specimens

Cast tensile and compression specimens were prepared from each aged resin lot using a silicon mold. The resin was dispensed into the mold and cured under 470 nm illumination for the prescribed exposure time. Figure A2a,b show the casting process for the compression samples and the resulting cast parts after post-processing. Additionally, Figure A2c displays the volumetrically printed compression parts fabricated with *Aged_0d* and *Aged_12d* resins, visually highlighting the dimensional variation and over-polymerization resulting from thermal aging. Figure A2d shows the printed tensile samples, which illustrate the visible stitching line at the boundary of the projection fields generated by the dual-projector upscaling setup.

### Appendix A.5. Experimental Concept and Workflow

Figure A3 illustrates the experimental concept and workflow used to evaluate the effect of thermal aging on VAM resins, including both printed and cast parts as control groups.
